# Clinical outcomes of first-line combination therapy with immune checkpoint inhibitor for metastatic non-clear cell renal cell carcinoma: a multi-institutional retrospective study in Japan

**DOI:** 10.1007/s10147-024-02612-1

**Published:** 2024-09-02

**Authors:** Akihiro Yoshimura, Taigo Kato, Yasutomo Nakai, Masao Tsujihata, Shingo Toyoda, Mototaka Sato, Kyosuke Matsuzaki, Wataru Nakata, Tetsuya Takao, Syunsuke Inoguchi, Yohei Okuda, Gaku Yamamichi, Yu Ishizuya, Yoshiyuki Yamamoto, Koji Hatano, Atsunari Kawashima, Shingo Takada, Hitoshi Inoue, Kensaku Nishimura, Osamu Miyake, Kazutoshi Fujita, Masashi Nakayama, Kazuo Nishimura, Norio Nonomura

**Affiliations:** 1 Departments of Urology, Osaka University Graduate School of Medicine, 2-2 Yamadaoka, Suita, Osaka 565-0871 Japan; 2 Department of Urology, Osaka International Cancer Institute, Osaka, Japan; 3 Department of Urology, Osaka Rosai Hospital, Sakai, Japan; 4 Department of Urology, Kindai University Hospital, Osakasayama, Japan; 5 Department of Urology, Toyonaka Municipal Hospital, Toyonaka, Japan; 6 Department of Urology, Osaka National Hospital, Osaka, Japan; 7 Department of Urology, Ikeda City Hospital, Ikeda, Japan; 8 Department of Urology, Osaka General Medical Center, Osaka, Japan; 9 Department of Urology, Osaka Police Hospital, Osaka, Japan

**Keywords:** Renal cell carcinoma, Non-clear cell renal cell carcinoma, Immune checkpoint inhibitor, Immune-related adverse event

## Abstract

**Background:**

In metastatic clear cell renal cell carcinoma (ccRCC), recent studies have shown promising efficacy of immune checkpoint inhibitor (ICI) combination therapy. However, there are insufficient evidences about clinical efficacy and safety of ICI combination therapy in metastatic non-ccRCC (nccRCC).

**Methods:**

We retrospectively investigated 44 patients treated with nivolumab plus ipilimumab (ICI + ICI group) or anti-PD-1/PD-L1 inhibitor plus tyrosine kinase inhibitors (TKI) (ICI + TKI group), and assessed clinical efficacy in both groups.

**Results:**

Of all patients, overall response rate and disease control rate for ICI combination treatments were 36.3% and 75%, respectively. The median progression-free survival (PFS) and overall survival (OS) was 8.8 and 23.9 months, respectively. Multivariate analysis revealed that the presence of liver metastasis significantly affected worse PFS and OS (*p* = 0.035 and *p* = 0.049). Importantly, PFS and OS seemed similar in ICI + ICI group and ICI + TKI group (*p* = 0.778 and *p* = 0.559). Although the discontinuation rate of the combination therapy due to adverse effects in patients aged ≥ 75 years was significantly higher compared to that in patients aged < 75 years (45% versus 12%, *p* = 0.017), there were no significant differences in PFS and OS between two groups (*p* = 0.290 and *p* = 0.257, respectively).

**Conclusion:**

This study confirms clinical benefit of ICI combination therapy for metastatic nccRCC patients in real-world settings. Furthermore, the effectiveness of combination therapy was comparable between patients aged < 75 and those ≥75 years with respect to clinical prognosis.

**Supplementary Information:**

The online version contains supplementary material available at 10.1007/s10147-024-02612-1.

## Introduction

Renal cell carcinoma (RCC) accounts for about 2–4% of all types of cancer worldwide [1]. The 5-year cancer specific survival rate in the early stage is reported to be approximately 90%, however, the 5-year overall survival plummets to 14% when patients once develop metastasis sites [2, 3]. In addition, 25–30% of RCC patients present with the evidence of distant metastasis at initial diagnosis, leading to the poor prognosis for patients in the advanced stage [4]. Non-clear cell renal cell carcinoma (nccRCC) accounts for approximately 25% of RCC cases and encompasses various histologic subtypes of RCC, including papillary, chromophobe, and translocation [5, 6]. So far, treatments in patients with nccRCC, including cytokine therapies, mTOR inhibitors, tyrosine-kinase inhibitors (TKIs), and immune checkpoint inhibitors (ICIs), have either not significantly enhanced clinical outcomes and have not been established as standard care practice [7–15].

Phase III clinical trials investigating the efficacy of ICIs combination therapies for advanced RCC have been conducted primarily in clear cell renal cell carcinoma (ccRCC) patients due to its high prevalence and the treatment paradigm for advanced nccRCC remains suboptimal, underscoring the importance of enrolling nccRCC patients in clinical trials. Recently, given these issues, several clinical trials showed durable antitumor activity in metastatic nccRCC patients with ICI plus TKI combination therapies [16, 17]. However, so far, only a few data are available on the clinical efficacy in metastatic nccRCC patients with ICI combination therapies in clinical settings.

In the present study, we aimed to report the real-world efficacy and safety of ICI combination therapies in Japanese patients with untreated metastatic nccRCC. We demonstrated the favorable response and feasible adverse effects for metastatic nccRCC patients treated with ICI combination therapies. Collectively, ICI combination therapies are considered as an effective and safe treatment for nccRCC in the real-world settings.

## Materials and methods

### Patients

We retrospectively investigated data from 44 metastatic nccRCC patients initially treated by ICI combination therapies at 9 institutions from July 2017 to July 2023. For each patient, we collected baseline demographic and clinical data including age, gender, the presence of nephrectomy, histological type based on the World Health Organization (WHO) 2022 classification of renal neoplasms, Karnofsky Performance Status, International Metastatic RCC Database Consortium (IMDC) risk classification, metastatic site, and the treatment details.

Tumor response was evaluated every 8–12 weeks, according to the Response Evaluation Criteria in Solid Tumors version 1.1 (RECIST ver1.1), using computed tomography. Adverse events (AEs) were evaluated by Common Terminology Criteria for AEs version 5.0 (CTCAE ver5.0). For each patient, the best response during treatment including complete response (CR), partial response (PR), stable disease (SD) or progressive disease (PD), was measured. Progression-free survival (PFS) was defined as the time from the initiation of ICIs to documented progression or death of any cause. Overall survival (OS) was defined as the time from the start of initial treatment to documented death of any cause or last contact.

The study was approved by the Institutional Review Board of each institution (approval number 23327 in Osaka University Hospital) and was conducted in accordance with the Declaration of Helsinki.

### Treatment

Patients were treated with nivolumab plus ipilimumab (ICI + ICI group) or anti-PD-1/PD-L1 inhibitor plus TKI (ICI + TKI group) according to the product information. The treatment was selected based on physician’s choice at each facility. Patients received therapy until either disease progression, clinical deterioration, unacceptable toxicity, or patient refusal.

### Statistical analysis

Differences between the two groups, patients in ICI + ICI group and those in ICI + TKI group were compared using Fisher’s exact tests for categorical variables. PFS and OS were estimated by the Kaplan–Meier method and compared with the log-rank test. The prognostic significance of certain parameters was assessed by the Cox proportional hazards regression model. Differences were considered significant at *p* value <0.05. All statistical analyses were conducted in JMP-software ver. 17.0 (SAS Institute, Cary, NC, USA).

## Results

### Patients’ characteristics

The clinical characteristics of all patients are shown in Table 1. The median age at treatment was 64 years (range, 29–86). The most predominant histological type was papillary RCC (43.2%), followed by translocation RCC (13.6%), and then mucinous tubular and spindle RCC (9.1%). With respect to the IMDC risk classification, 9%, 57%, and 34% were classified as favorable-, intermediate-, and poor-risk, respectively. Nephrectomy had been performed in 24 patients (54.5%). The most common sites of metastasis were lymph nodes in 25 cases (56.8%), followed by lung in 20 cases (45.5%), bone in 12 cases (27.3%), and liver in 11 cases (25.0%) (Supplementary Table 1).

**Table 1:** Baseline characteristics of nccRCC patients

	All (*n* = 44)	ICI + TKI (*n* = 24)	ICI + ICI (*n* = 20)	*p* value
Age (years, median) (range)	64 (29–86)	65 (29–86)	64 (32–83)	0.659
Gender (female/male)	7/37	4/20	3/17	0.880
Histological type				
Papillary	19 (43.2%)	9 (37.5%)	10 (50.0%)	0.405
Chromophobe	3 (6.8%)	3 (12.5%)	0	0.101
Collecting duct	2 (4.5%)	2 (8.3%)	0	0.186
Translocation	6 (13.6%)	4 (16.7%)	2 (10.0%)	0.521
ACD-associated	3 (6.8%)	3 (12.5%)	0	0.101
Mucinous tubular and spindle	4 (9.1%)	0	4 (20.0%)	0.022
Unclassified	7 (15.9%)	3 (12.5%)	4 (20.0%)	0.498
IMDC risk				
Favorable	4 (9.1%)	4 (16.7%)	0	0.056
Intermediate	25 (56.8%)	12 (50.0%)	13 (65.0%)	0.317
Poor	12 (27.3%)	8 (33.3%)	7 (35.0%)	0.908
Nephrectomy				
Yes/no	24/20	14/10	10/10	0.580
Metastasis				
Single/multiple	29/15	18/6	11/9	0.163
1st-line				
Pem + Axi	7 (15.9%)	7 (29.2%)	–	
Nivo + Cabo	5 (11.4%)	5 (20.8%)	–	
Ave + Axi	8 (18.2%)	8 (33.3%)	–	
Pem + Len	4 (9.1%)	4 (16.7%)	–	
Ipi + Nivo	20 (45.5%)	–	20 (100%)	
2nd-line				
Cabo	16 (36.4%)	10 (41.6%)	6 (30.0%)	0.423
Axi	5 (11.4%)	1 (4.2%)	4 (20.0%)	0.099
Sni	1 (2.3%)	1 (4.2%)	0	0.356
Pazo	1 (2.3%)	0	1 (5.0%)	0.268
Nivo	1 (2.3%)	1 (4.2%)	0	0.356

*Axi* axitinib, *Ipi* ipilimumab, *Nivo* nivolumab, *Cabo* cabozantinib, *Ave* avelumab, *Len* lenvatinib, *Sni* sunitinib, *Pazo* pazopanib

### Clinical outcomes of metastatic nccRCC patients with combination therapy

**Fig. 1:**
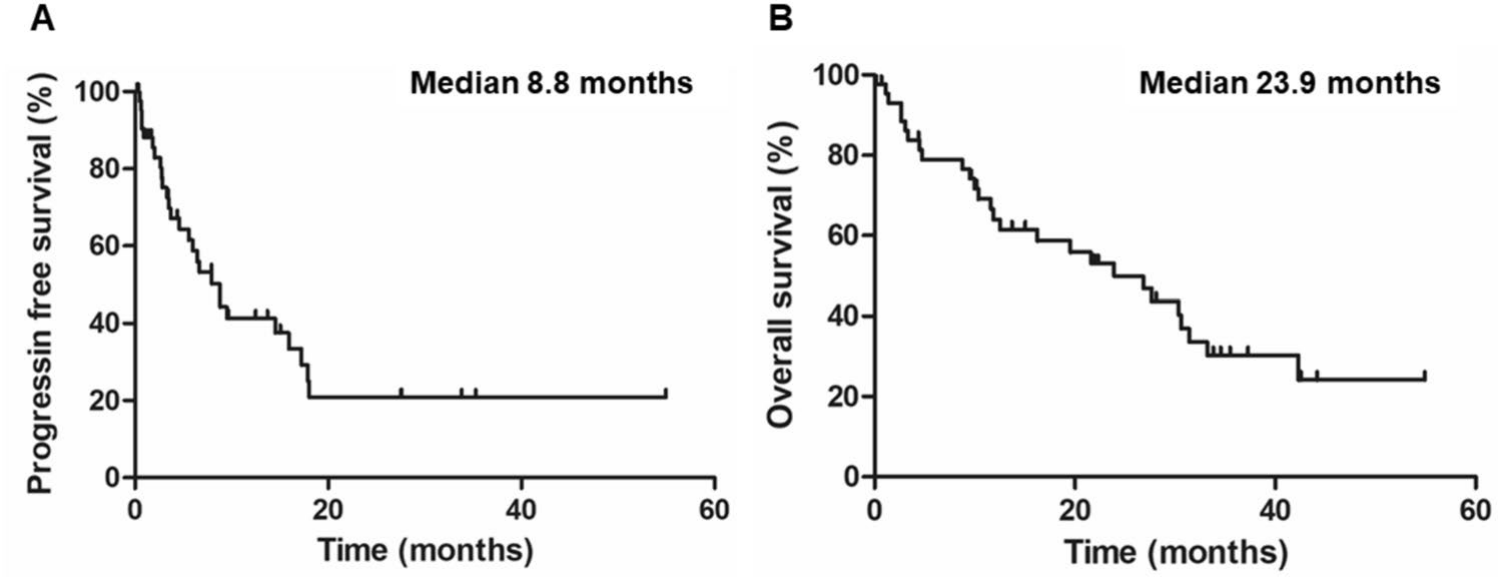
Kaplan–Meier survival curves for all metastatic nccRCC patients treated with ICI combination therapy. **A** Progression-free survival (PFS). **B** Overall survival (OS)

**Fig. 2:**
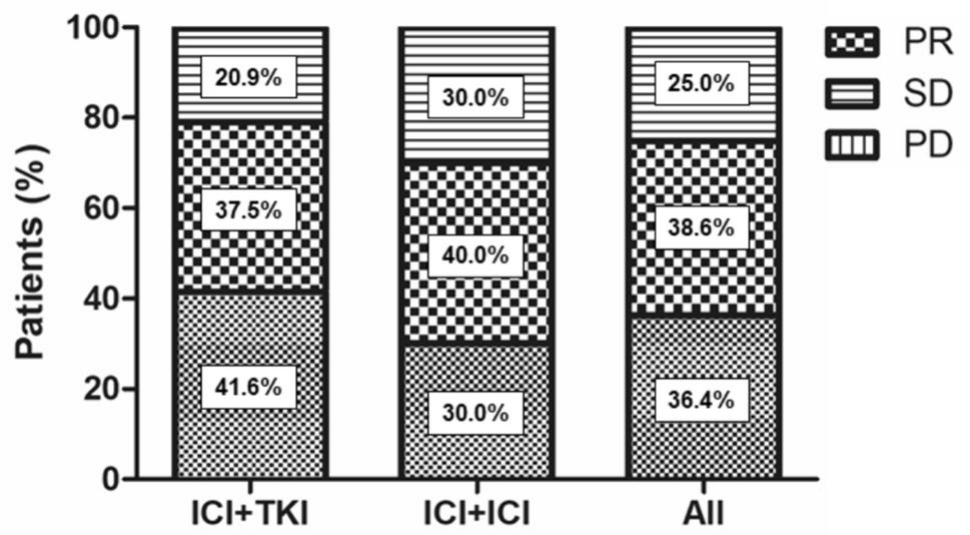
Response rates in nccRCC patients with ICI combination therapy. There were no patients with CR in this study. The ORR of ICI + TKI group, ICI + ICI group and all cases was 41.6%, 30.0% and 36.3%, respectively. The DCR was 79.2%, 70.0% and 75.0%, respectively

The median duration of first-line ICI combination treatments was 5.8 month (range, 0.2–54.9), and the median follow-up period was 15.2 month (range, 0.2–54.9). During the observational period, 27 patients (61.4%) died from cancer. Overall, the median OS was 23.9 months, and the median PFS was 8.8 months (Fig. 1). Among 44 patients, CR and PR were achieved in 0 and 16 patients (36.3%) and SD was observed in 17 patients (38.6%), resulting in objective response rate (ORR) of 36.3% and disease control rate (DCR) of 75.0% (Fig. 2).

**Fig. 3:**
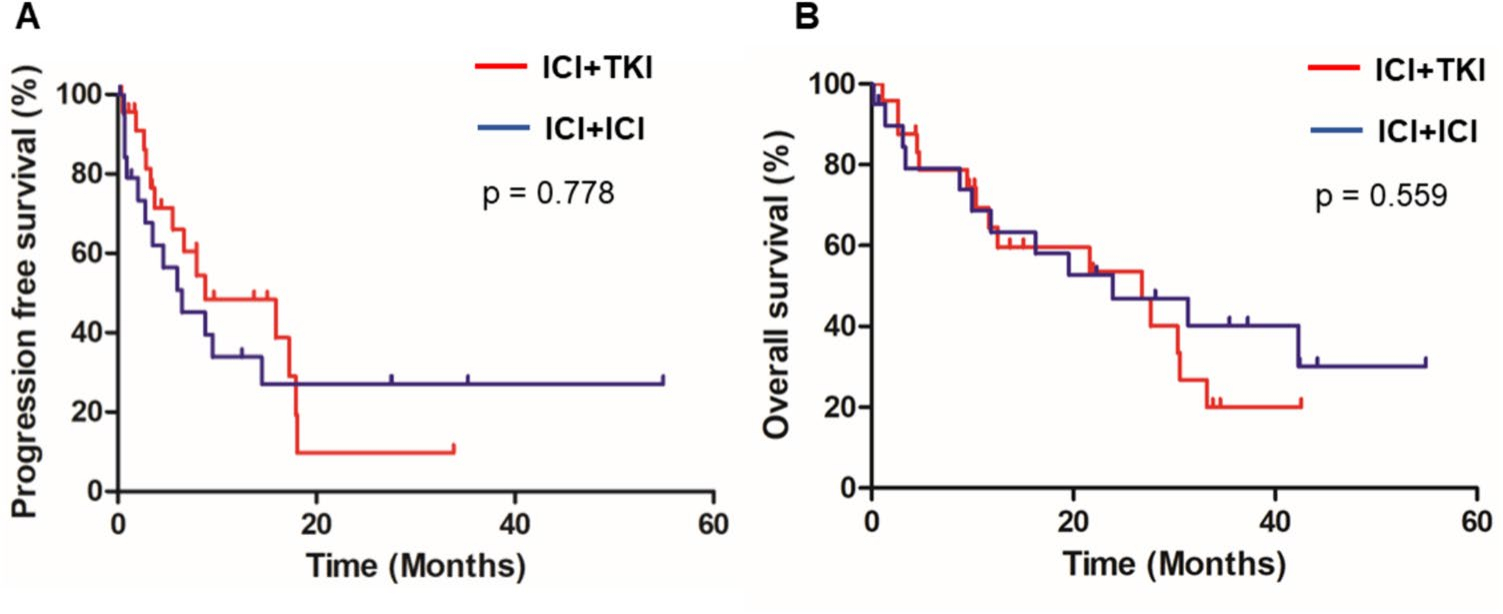
Survival analysis for patients nivolumab plus ipilimumab and anti-PD-1/PD-L1 inhibitor plus tyrosine kinase inhibitors. Kaplan–Meier survival curves showing **A** progression-free survival (PFS) and **B** overall survival (OS) between nivolumab plus ipilimumab (ICI + ICI group) and anti-PD-1/PD-L1 inhibitor plus TKI (ICI + TKI group). PFS and OS were similar in both groups (8.8 months versus 6.4 months, *p* = 0.778 and 26.8 months versus 23.9 months, *p* = 0.559, respectively)

Next, we investigated the regimen of ICIs combination therapies affected clinical outcomes in our cohort. Importantly, there was no significant difference in PFS and OS between ICI + TKI and ICI + ICI groups (*p* = 0.778 and *p* = 0.559, respectively) (Fig. 3). Even when limited to the patients with papillary RCC, there was no statistically significant difference between two groups in terms of PFS and OS (*p* = 0.556 and *p* = 0.676, respectively) (Supplementary Fig. 1).

Since there were no patients with favorable risk in the ICI + ICI group, we performed the additional analysis after excluding patients with favorable risk in the ICI + TKI group. As a result, there was no significant difference in PFS and OS between two groups (*p* = 0.776 and *p* = 0.433, respectively) (Supplementary Fig. 2).

### Clinical outcomes of patients with cabozantinib immediately after combination therapy as the second-line therapy

We further aimed to evaluate the efficacy of TKIs immediately after discontinuation of ICIs combination therapies. As shown in Table 1, the most common treatment after the combination therapies was cabozantinib (36.3%). The median PFS was 6.8 months in these patients (Supplementary Fig. 3). Among them, CR and PR were achieved in 1 (6.3%) and 2 patients (12.5%), and SD was observed in 6 patients (37.5%), resulting in the ORR of 18.8% and the DCR of 56.3% (Supplementary Table 2). In contrast, in patients with other types of TKIs, the ORR and DCR were 14.3% and 28.5%, respectively.

### Safety analysis

**Fig. 4:**
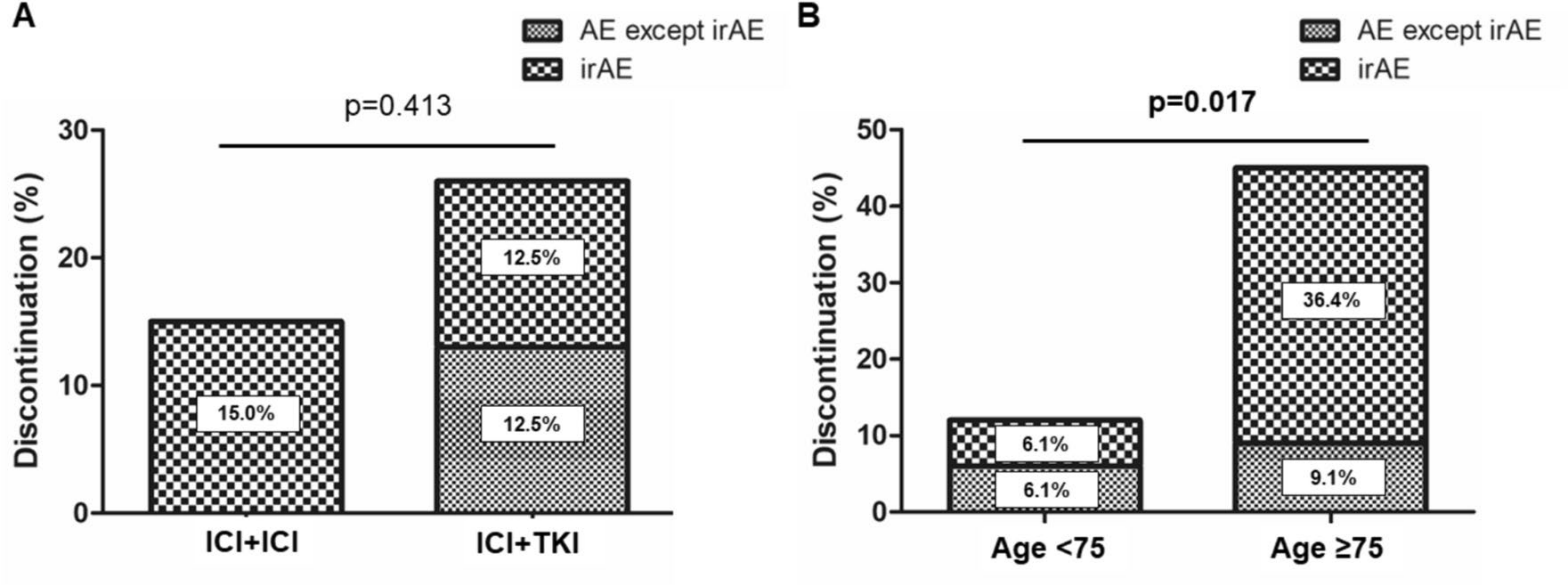
Percentage of patients who discontinued treatment because of adverse events. **A** There was no significant difference in the percentage of discontinuations due to adverse events (AEs) between the ICI + ICI group and the ICI + TKI groups (15.0% versus 25.0%, *p* = 0.413). **B** The discontinuation rate of treatment due to AEs in patients aged ≥ 75 years was significantly higher compared to that in patients aged < 75 years (45.5% versus 12.2%, *p* = 0.017). irAE; immune-related adverse event

Specific details regarding the AEs reported in our study are shown in Table 2. In the present study, any grade and grade 3–5 immune-related AEs (irAEs) occurred in 16 (36.3%) and 9 (20.5%) patients. Two patients with pneumonitis experienced grade 5 irAE. They were treated with nivolumab plus cabozantinib. The most frequent irAEs were related to hepatitis (13.6%) followed by adrenal insufficiency (9.0%) and pneumonitis (9.0%). All irAEs except grade 5 were recovered, although 4 patients with adrenal insufficiency required continuous corticosteroid therapy. The most common AE caused by TKI was fatigue (20.8%), followed by hand-foot syndrome (12.5%) and hepatitis (12.5%). All patients with AEs induced by TKIs showed improvement upon discontinuation of the medication. During the observational period, 9 patients discontinued the combination therapy due to the occurrence of AEs. Importantly, there was no significant difference in the percentage of discontinuations due to AEs between the ICI + ICI group and the ICI + TKI groups (15.0% versus 25.0%, *p* = 0.413, Fig. 4A).

**Table 2:** Summary of AEs in nccRCC patients with ICI combination therapy

	Grade 1	Grade 2	Grade 3	Grade 4	Grade 5
irAE ICI + ICI group (*n* = 20)					
Hepatitis		2	1		
Pneumonitis		1	1		
Adrenal insufficiency		2			
Fever	1	1			
Hypothyroidism		2			
Thrombocytopenia			1		
Hypertension		1			
Hoarseness	1				
Total	2 (10.0%)	9 (45.0%)	3 (15.0%)	0	0
irAE ICI + TKI group (*n* = 24)					
Hepatitis		1	1	1	
Pneumonitis					2
Adrenal insufficiency		1	1		
Cytokine release syndrome				1	
Total	0	2 (8.3%)	2 (8.3%)	2 (8.3%)	2 (8.3%)
AEs of TKI ICI + TKI group (*n* = 24)					
Fatigue	2		3		
Hand-foot syndrome	3				
Rash	2				
Hoarseness		1			
Hypothyroidism	1				
Hepatitis			3		
Total	8 (33.3%)	1 (4.2%)	6 (25.0%)	0	0

Recently, the efficacy and safety of ICI combinations therapy in elderly patients with ccRCC have been reported [18, 19]. In our cohort, 25.0% of patients were aged ≥ 75 years. When patients were stratified into two groups according to their age (≥75 years or <75 years, *n* = 11 and *n* = 33), the incidence of AEs did not differ between the two groups (63.6% versus 48.5%, *p* = 0.384). Although the discontinuation rate of the combination therapy due to AEs in patients aged ≥ 75 years was significantly higher compared to that in patients aged < 75 years (45.5% versus 12.2%, *p* = 0.017) (Fig. 4B), there were no significant differences in PFS and OS between two groups (*p* = 0.290 and *p* = 0.257, respectively) (Supplementary Fig. 4).

### Correlation between the clinicopathologic factors and clinical efficacy

We further investigated the impacts of several clinicopathologic factors on PFS and OS in metastatic nccRCC patients with ICIs combination therapy (Table 3 and Table 4). Univariate analysis revealed that female gender and liver metastasis were significantly associated with the decrease of PFS. Interestingly, multivariate analysis also demonstrated that female and the presence of liver metastasis were both significantly associated with worse PFS. With respect to OS, univariate and multivariate analysis also revealed that the presence of liver metastasis had significant association with worse OS.

**Table 3:** Univariate and multivariate Cox proportional hazards regression analysis for the prognostic factors of PFS (*n* = 44)

Valuables	Univariate analysis		Multivariate analysis	
	HR (95%CI)	*p*	HR (95%CI)	*p*
Age				
<75	Reference			
≥75	0.595 (0.225–1.574)	0.295	–	–
Gender				
Male	Reference			
Female	2.442 (1.016–5.870)	0.046	2.691 (1.105–6.551)	0.029
IMDC				
≤Intermediate	Reference			
Poor	1.720 (0.794–3.725)	0.169	–	–
Histological type				
Papillary	Reference			
Other	0.839 (0.393–1.793)	0.652	–	–
Metastatic site				
Single	Reference			
Multiple	1.229 (0.550–2.746)	0.615	–	–
Liver metastasis				
No	Reference			
Yes	2.493 (1.015–6.122)	0.046	2.670 (1.067–6.680)	0.035
Baseline CRP				
<1	Reference			
≥1	0.814 (0.369–1.793)	0.601	–	–
irAE				
No	Reference			
Yes	0.698 (0.313–1.560)	0.381	–	–

**Table 4:** Univariate and multivariate Cox proportional hazards regression analysis for the prognostic factors of OS (*n* = 44)

Valuables	Univariate analysis		Multivariate analysis	
	HR (95%CI)	*p*	HR (95%CI)	*p*
Age				
<75	Reference			
≥75	1.584 (0.709–3.538)	0.261	–	–
Gender				
Male	Reference			
Female	1.403 (0.529–3.721)	0.496	–	–
IMDC				
≤Intermediate	Reference			
Poor	1.720 (0.794–3.725)	0.169	–	–
Histological type				
Papillary	Reference			
Other	1.508 (0.703–3.237)	0.292	–	–
Metastatic site				
Single	Reference			
Multiple	2.035 (0.908–4.558)	0.084	1.528 (0.614–3.803)	0.362
Liver metastasis				
No	Reference			
Yes	3.091 (1.334–7.162)	0.008	2.555 (1.002–6.513)	0.049
Baseline CRP				
<1	Reference			
≥1	1.496 (0.697–3.212)	0.302	–	–
irAE				
No	Reference			
Yes	0.690 (0.315–1.514)	0.302	–	–

## Discussion

The landscape of oncology has been dramatically transformed by the emergence of ICIs, greatly improving the prognosis of cancers that were previously deemed incurable [20]. However, the therapeutic efficacy of ICIs for metastatic nccRCC remains poorly understood. An analysis of previously untreated metastatic nccRCC patients with pembrolizumab monotherapy reported a notable efficacy with an ORR of 26.7% across all patients [21]. Most recently, the KEYNOTE B61 trial demonstrated promising outcomes, with an ORR of 49%, 12-month PFS of 63%, and 12-month OS of 82% with the combination of pembrolizumab plus lenvatinib [16]. Despite these promising results in clinical trials, real-world evidences supporting the effect of ICIs combination therapy for nccRCC is still limited, underscoring the necessity for further studies to validate its efficacy. Hence, in this study, we conducted the data analysis of metastatic nccRCC patients treated with ICIs combination therapy in real-world settings and clarified some evidences affecting clinical outcomes.

First, we demonstrated favorable clinical outcome with ICI combination therapies as evidenced by the high ORR of 36.3% and DCR of 75.0% (Fig. 2). Moreover, PFS and OS were similar in ICI + TKI and ICI + ICI groups (Fig. 3). Our data partially align with those reported by Teishima et al. [22] reported that described the ORR was 33.3% and the median PFS in the ICI + TKI group was significantly longer than that in the ICI + ICI group (9.7 months and 4.6 months, *p* = 0.049), although there was no significant difference in OS between these groups (*p* = 0.398). Importantly, liver metastasis significantly affected worse PFS and OS in our cohort, which is considered a poor efficacy and prognostic factor [23], providing physicians the information that they may use the second-line cabozantinib which was shown to be effective in treating patients with liver metastasis [24]. Furthermore, we first confirmed that the effectiveness of ICIs combination therapies was comparable between metastatic nccRCC aged ≥ 75 and <75 years with respect to their PFS and OS. So far, several studies reported that first-line ICIs combination therapies showed favorable efficacy across age groups in patients with ccRCC [18, 19]. Further prospective studies will be needed to confirm the benefit and risks of ICIs combination therapies in elderly patients with nccRCC.

Secondary, we confirmed that the effect of cabozantinib immediately after ICI discontinuation was promising, as evidenced by the high ORR (18.8%) and DCR (56.3%) (Supplementary Table 2), leading to the clinical benefit of long PFS (6.8 months, Supplementary Fig. 3). Our findings partially align with those reported by Pal et al. [25] who described papillary RCC patients treated with cabozantinib as first-line therapy demonstrating an ORR of 23% and a median PFS of 9.0 months (95%CI 5.6–12.4) in SWOG1500 trial. Martínez Chanzá et al. [26] also reported the clinical outcomes of cabozantinib during any treatment line, which showed ORRs of 27% (30 of 112 patients, 95% CI 19–36), and median PFS and OS of 7.0 and 12.0 months, respectively across all histologies of nccRCC. They also characterized the mutational landscape of tumor DNA in 54 patients with available next-generation sequencing data, and found that a certain number of patients with somatic mutations in *CDKN2A* or *MET* (22% and 20%, respectively) tended to have favorable response to cabozantinib. Such an analysis including circulating tumor DNA would help to predict the clinical response to TKI therapy in mRCC patients after discontinuation of ICIs.

There are several limitations to this study. Firstly, the present study is observational and retrospective study with a small sample size due to the limited number of patients with nccRCC. Secondly, given that nccRCC includes various histologic types with distinct genetic backgrounds and molecular characteristics, a greater accumulation of precise pathological diagnoses through immunohistologic or genetic analyses is necessary to establish personalized treatment for nccRCC.

## Conclusion

In conclusion, to our knowledge, the present study has the longest duration and is based on the largest population of metastatic nccRCC patients treated with ICIs combination therapy in Japan to date. These findings endorse the utilization of ICI combination therapy as a primary treatment option for patients with advanced nccRCC, irrespective of histologic subtype. In addition, considering that the liver metastasis was an independent prognostic factor in nccRCC patients, it is important to switch to the second-line therapy immediately after the first-line treatment failure.

## Supplementary Information

Below is the link to the electronic supplementary material.

**Supplementary Fig. 1.** Kaplan–Meier survival curves showing (A) progression-free survival and (B) overall survival of papillary RCC patients between ICI + ICI group (*n* = 10) and ICI + TKI groups (*n* = 9) (9.2 months *versus* 5.6 months, *p* = 0.559 and 31.4 months versus 27.6 months, *p* = 0.676, respectively). **Supplementary Fig. 2.** Kaplan–Meier survival curves showing (A) progression-free survival and (B) overall survival of nccRCC patients excluding favorable risk between ICI + ICI group (*n* = 20) and ICI + TKI groups (*n* = 20) (6.4 months *versus* 8.8 months, *p* = 0.776 and 23.9 months versus 21.6 months, *p* = 0.433, respectively). **Supplementary Fig. 3.** Kaplan–Meier survival curves showing progression-free survival of metastatic nccRCC patients treated with second-line cabozantinib (*n* = 16, median 16.1 months). **Supplementary Fig. 4.** Kaplan–Meier survival curves showing (A) progression-free survival and (B) overall survival of metastatic nccRCC patients between the patients aged ≥ 75 years and aged < 75 years (14.5 months versus 6.4 months, *p* = 0.290 and 16.2 months versus 26.8 months, *p* = 0.257, respectively). (PDF 424 KB)
