# Supplementary material for: Clinical outcomes of first-line combination therapy with immune checkpoint inhibitor for metastatic non-clear cell renal cell carcinoma: a multi-institutional retrospective study in Japan
Source: Int J Clin Oncol. 2024 Sep 2;29(12):1916–24. doi: 10.1007/s10147-024-02612-1 (PMC11588831; doi:10.1007/s10147-024-02612-1)
Supplement: Supplementary file 1 — Supplementary Fig. 1. Kaplan–Meier survival curves showing (A) progression-free survival and (B) overall survival of papillary RCC patients between ICI + ICI group (n = 10) and ICI + TKI groups (n = 9) (9.2 months versus 5.6 months, p = 0.559 and 31.4 months versus 27.6 months, p = 0.676, respectively). Supplementary Fig. 2. Kaplan–Meier survival curves showing (A) progression-free survival and (B) overall survival of nccRCC patients excluding favorable risk between ICI + ICI group (n = 20) and ICI + TKI groups (n = 20) (6.4 months versus 8.8 months, p = 0.776 and 23.9 months versus 21.6 months, p = 0.433, respectively). Supplementary Fig. 3. Kaplan–Meier survival curves showing progression-free survival of metastatic nccRCC patients treated with second-line cabozantinib (n = 16, median 16.1 months). Supplementary Fig. 4. Kaplan–Meier survival curves showing (A) progression-free survival and (B) overall survival of metastatic nccRCC patients between the patients aged ≥ 75 years and aged < 75 years (14.5 months versus 6.4 months, p = 0.290 and 16.2 months versus 26.8 months, p = 0.257, respectively). (PDF 424 KB) [file 10147_2024_2612_MOESM1_ESM.pdf]

Supplementary data

Title: Clinical outcomes of first-line combination therapy with immune checkpoint inhibitor for metastatic non-clear cell renal cell carcinoma: A multi-institutional retrospective study in Japan

Journal: International Journal of Clinical Oncology

Akihiro Yoshimura[1], Taigo Kato[1*], Yasutomo Nakai[2], Masao Tsujihata[3], Shingo Toyoda[4], Mototaka Sato[5], Kyosuke Matsuzaki[6], Wataru Nakata[7], Tetsuya Takao[8], Syunsuke Inoguchi[9], Yohei Okuda[1], Gaku Yamamichi[1], Yu Ishizuya[1], Yoshiyuki Yamamoto[1], Koji Hatano[1], Atsunari Kawashima[1], Shingo Takada[9], Hitoshi Inoue[7], Kensaku Nishimura[6], Osamu Miyake[5], Kazutoshi Fujita[4], Masashi Nakayama[2], Kazuo Nishimura[2], Norio Nonomura[1]

1 Departments of Urology, Osaka University Graduate School of Medicine, Suita, Japan
2 Department of Urology, Osaka International Cancer Institute, Osaka, Japan
3 Department of Urology, Osaka Rosai Hospital, Sakai, Japan
4 Department of Urology, Kindai University Hospital, Osaka-sayama, Japan
5 Department of Urology, Toyonaka Municipal Hospital, Toyonaka, Japan
6 Department of Urology, Osaka National Hospital, Osaka, Japan
7 Department of Urology, Ikeda City Hospital, Ikeda, Japan
8 Department of Urology, Osaka General Medical Center, Osaka, Japan
9 Department of Urology, Osaka Police Hospital, Osaka, Japan

E-mail of first author: yoshimura@uro.med.osaka-u.ac.jp (A. Yoshimura)

*Correspondence to: Taigo Kato, Departments of Urology, Osaka University Graduate School of Medicine, 2-2 Yamadaoka, Suita City, Osaka 565-0871, Japan
TEL: +81-06-6879-3531 Fax: +81-06-6879-3539
E-mail: kato@uro.med.osaka-u.ac.jp

Supplementary Figure 1

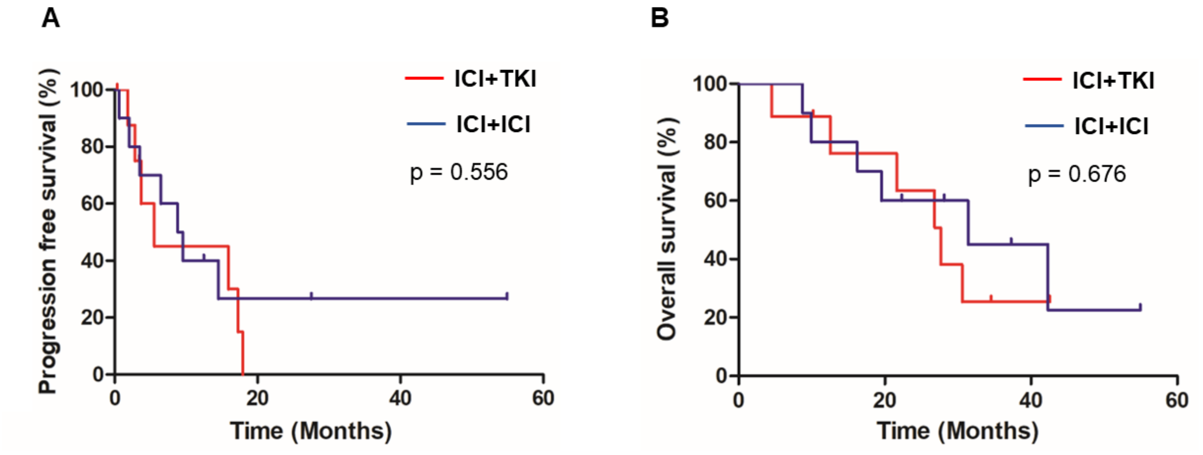

Supplementary Figure 2

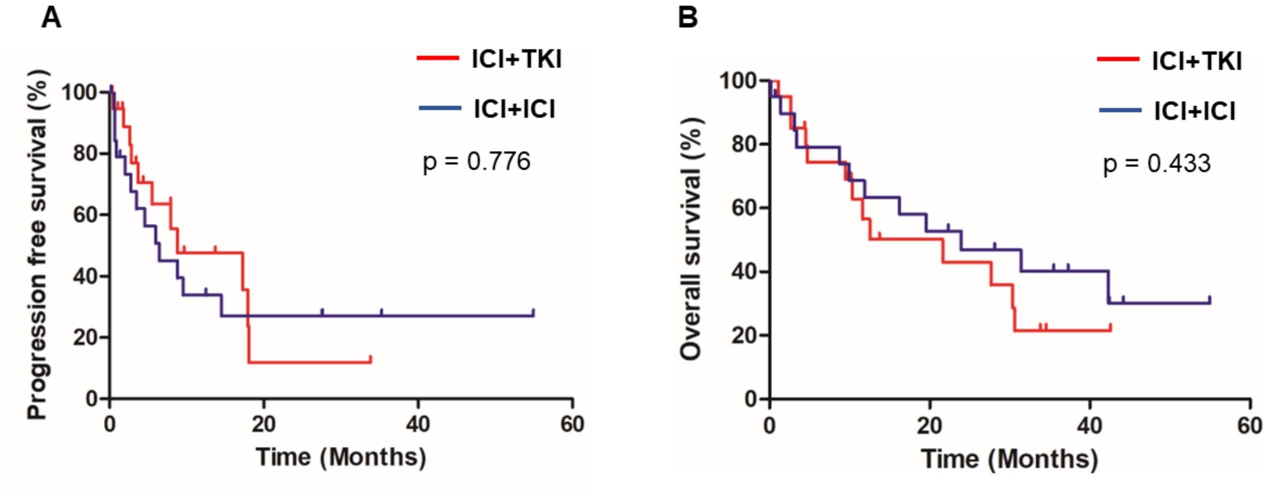

Supplementary Figure 3

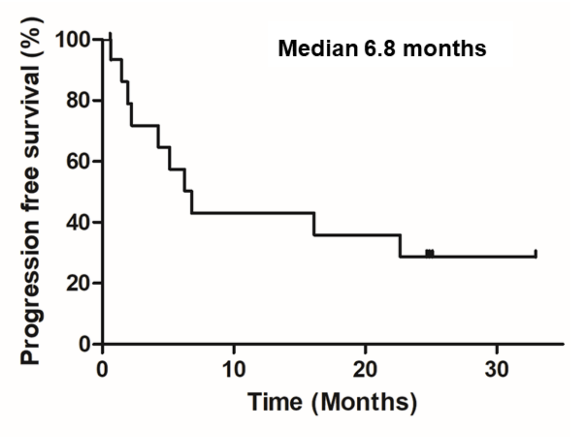

Supplementary Figure 4

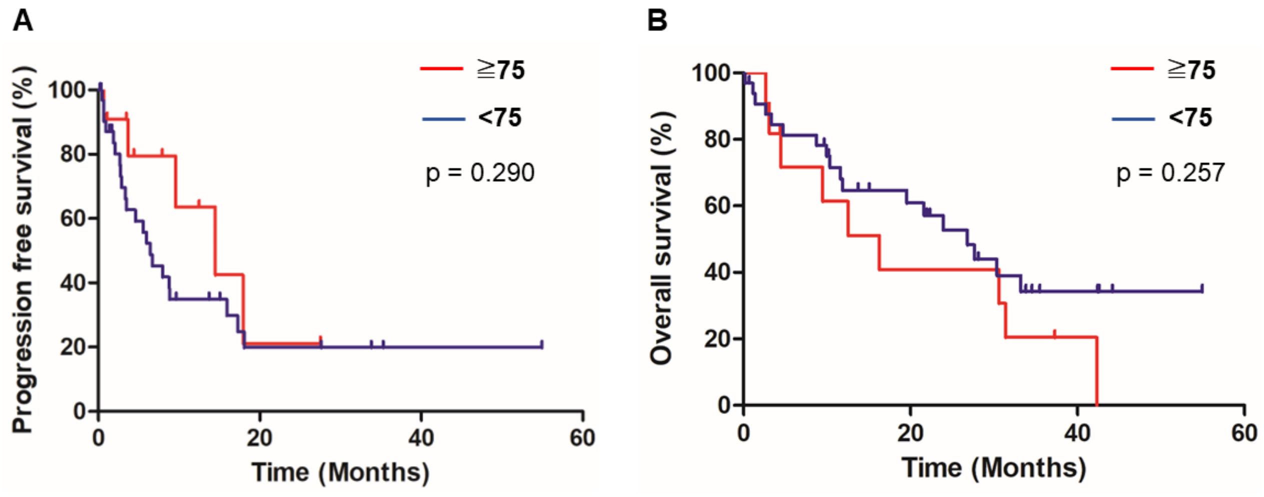

Supplementary Table 1. Summary of metastatic sites.

**Metastatic site**	**n=44**
Lung	20 (45.4 %)
Bone	12 (27.2 %)
Liver	11 (25.0 %)
Peritoneum	4 (9.1 %)
Adrenal gland	2 (4.5 %)
Pleura	2 (4.5 %)
Ureter	1 (2.3 %)
Lymph node	25 (56.8 %)

Supplementary Table 2. Response rates in nccRCC patients with second-line TKI therapy.

	**Cabozantinib**<br>**(n = 16)**	**Other TKI**<br>**(n = 7)**	**TKI All**<br>**(n = 23)**
Best response, n (%)			
CR	1 (6.3)	0 (0)	1 (0)
PR	2 (12.5)	1 (14.3)	3 (13.0)
SD	6 (37.5)	1 (14.3)	7 (30.4)
PD	4 (25.0)	3 (21.4)	7 (30.4)
Unknown	3 (18.8)	2 (28.5)	5 (21.7)
ORR, n (%)	3 (18.8)	1 (14.3)	4 (17.4)
DCR, n (%)	9 (56.3)	2 (28.5)	11 (47.8)
